# Lentiviral expression of anti-microRNAs targeting miR-27a inhibits proliferation and invasiveness of U87 glioma cells

**DOI:** 10.3892/mmr.2012.915

**Published:** 2012-05-15

**Authors:** SU Y. FENG, CHANG G. DONG, WILLIAM K.K. WU, XIAO J. WANG, JIAN QIAO, JUN F. SHAO

**Affiliations:** 1 Department of Neurosurgery, Wuxi People’s Hospital of Nanjing Medical University, Wuxi 214023; 2 Department of Pathophysiology, College of Veterinary Medicine, China Agricultural University, Beijing 100094; 3 Institute of Digestive Disease, Department of Medicine and Therapeutics and LKS Institute of Health Sciences, The Chinese University of Hong Kong, Hong Kong SAR, P.R. China

**Keywords:** microRNA, glioma, lentivirus, proteomics, proliferation

## Abstract

Glioma is a highly fatal malignant disease and its treatment options are limited. microRNAs represent a novel target for the treatment of cancer. In the present study, we used a lentiviral vector to stably express anti-microRNAs targeting the oncogenic miR-27a in U87 glioma cells. The stable expression of anti-miR-27a significantly reduced the proliferation and increased the accumulation of U87 cells in the sub-G1 phase as determined by Cell Counting kit-8 (CCK-8) assays and flow cytometry, respectively. Results from the Matrigel Transwell assay also indicated that the inhibition of miR-27a substantially impaired the invasiveness of U87 cells. By combining bioinformatic and proteomic approaches, we identified the mRNAs of 8 proteins upregulated in anti-miR-27a-expressing U87 cells as putative direct targets of miR-27a. Collectively, these data suggest that the lentiviral expression of anti-miR-27a is a feasible approach for the suppression of malignant phenotypes of glioma cells.

## Introduction

Glioma, which incurs high mortality due to its fast-growing and invasive nature, is the most frequently encountered intracranial tumor (1). The pathogenesis of glioma is complex and involves the aberrant activation of proto-oncogenes (e.g., *EGFR* and *IDH1/2*) and inactivation of tumor suppressor genes (e.g., *TP53* and *PTEN*) (2–4). The causes and consequences of intracellular signaling network dysregulation in the development of glioma, however, have not yet been fully elucidated. A number of treatment modalities, such as neurosurgery, chemotherapy and radiotherapy, have been devised for gliomas (5). Nevertheless, the prognosis of patients with this malignancy remains dismal, with a median survival time of 9–12 months after diagnosis. This highlights the need to develop novel agents for the treatment of this highly aggressive disease.

microRNA (miRNA) is a class of non-coding RNA. These RNAs, which are of 20–25 nucleotides in length, carry out their biological functions by binding to the 3′ untranslated regions (UTRs) of their target mRNAs, thereby repressing the translation of target mRNAs into proteins and/or directly inducing the degradation of target mRNAs (6,7). miRNA genes are first transcribed to primary miRNAs, which are then processed by Drosha into precursor miRNAs. After exportation from the nucleus, precursor miRNAs are further processed by the RNase Dicer to produce miRNA:miRNA^*^ duplexes. The mature miRNA strand then guides the RNA-induced silencing complex to the target mRNA to repress its expression (7,8). miRNA is emerging as a novel player in tumorigenesis. In this regard, miRNA expression is dysregulated in most, if not all, types of cancer. In glioma tissues, the aberrant upregulation of miR-15b (9), miR-21 (10), miR-221/222 (11) and miR-296 (6), and the downregulation of miR-7 (12), miR-124 (13), miR-128 (14), miR-137 (15) and miR-181a/b/c (16,17) have been reported. Notably, the restoration of these dysregulated miRNAs to normal expression levels has been shown to impair the growth and survival of glioma cells. These findings support that miRNAs play important functional roles in cancer development. Thus, miRNA represents a novel target for the treatment of glioma.

In the present study, we employed a novel miRNA targeting approach using a lentiviral vector to deliver anti-miRNAs into glioma cells. This vector was constructed to produce short hairpin RNAs, which eventually give rise to short, single-stranded anti-miRNAs that competitively bind to and inhibit endogenous miR-27a, a miRNA that displays oncogenic properties in many types of solid tumors, including breast (18), gastric (19), colon (20) and pancreatic cancers (21). miR-27a has also been shown to be expressed in glioma cells and is detectable in glioma-secreted exosomes (22). In this study, we provide evidence that targeting miR-27a by the lentiviral expression of anti-miRNAs substantially impairs the malignant phenotypes of glioma cells. Moreover, coupled with computational prediction and proteomic analysis, we successfully identified the targets specifically repressed by miR-27a in glioma cells.

## Materials and methods

### Construction of short hairpin-expressing lentiviral vector

The short hairpin RNAs targeting miR-27a were cloned into the pGreenPuro™ shRNA expression lentivector (SBI). The hairpins were rationally designed for asymmetry such that the upper strand of the hairpin did not contain the miR-27a sequence, whereas the lower strand was fully complementary to miR-27a. The expression of the short hairpin was driven by the H1 promoter. This vector also encoded cop green fluorescence protein (copGFP) for the selection of stably transfected clones. Successful cloning was confirmed by sequencing. The pPACK-H1 Lentivector Packaging System (SBI) and the 293TN cell line (SBI) were used for the production of pseudoviral particles according to the manufacturer’s instructions. U87 cells were then transduced at a multiplicity of infection (MOI) of 3.

### Cell culture and proliferation assay

The U87 human glioma cell line was obtained from the American Type Culture Collection (Manassas, VA, USA). The cells were maintained in DMEM (Invitrogen, Carlsbad, CA, USA), supplemented with 10% fetal bovine serum (Thermo Scientific), 100 U/ml penicillin and 100 μg/ml streptomycin (Invitrogen) at 37°C in a humidified atmosphere of 5% CO_2_ and 95% air. Cell proliferation was measured by the colorimetric Cell Counting kit-8 (CCK-8) assay (Dojindo). The transfected cells were plated at a density of 5,000 cells/well in 96-well plates. After incubation for 2 h to allow cell attachment to the bottom of the well, 10 μl CCK-8 solution were added to each well at 0, 24 and 48 h, and the plates were incubated for another 2 h. The optical density was then determined at 450 nm using a microplate reader.

### Cell cycle analysis

U87 cells were fixed with ice-cold 70% ethanol in phosphate-buffered saline, followed by incubation with 50 μg/ml propidium iodide, 3.8 mmol/l sodium citrate and 0.5 μg/ml RNase A at 4°C for 3 h and analyzed by flow cytometry.

### Cell invasion assay

The invasive capacity of cells was determined using the BD BioCoat Matrigel invasion chambers (8-μm pores) (BD Biosciences). The transfected cells were seeded on the top chamber of each insert with complete medium added to the bottom chamber. After 48 h, cells on the membrane were wiped off with a cotton swab. Fixed and stained with H&E, cells on the underside of the membrane were counted from 4 microscope fields (magnification, ×200). The mean number of invading or migrating cells was expressed as a percentage relative to the control.

### Quantitation of miR-27a expression

Total RNA was isolated using TRIzol (Invitrogen) and cDNA was synthesized using the QuantiMir kit (SBI) following the manufacturer’s instructions. Real-time PCR was performed with miR-27a-specific forward primer and universal reverse primer. Conditions for real-time PCR were 50°C for 2 min, 95°C for 10 min, 40 cycles of 95°C for 15 sec and 60°C for 1 min. Quantitative PCR was carried out using SYBR-Green JumpStart Taq ReadyMix (Sigma) and the 7300 Real-Time PCR Detection System (ABI). The results were analyzed using the comparative threshold cycle (CT) method.

### Two-dimensional (2D) electrophoresis

The immobilized pH gradient (IPG) strip (pH 3–10, length 13 cm; Bio-Rad, Hercules, CA, USA) was rehydrated with 1,500 μg protein in 450 ml rehydration buffer containing 7 M urea, 2 M thiourea, 4% CHAPS, 65 mM DTT, 20 mM Trizma base, 1% IPG buffer and 0.002% bromophenol blue for 14 h at room temperature. Isoelectric focusing (IEF) was performed using the Protean IEF System (Bio-Rad) for a total of 70 kVh. The strip was then subjected to two-step equilibration in a buffer containing 6 M urea, 20% glycerol, 2% SDS and 50 mM Tris-HCl (pH 8.8), with 2% w/v DTT for the first step and 2.5% w/v iodoacetamide for the second step. The second-dimension SDS-PAGE (12% T, 260×200×1.5 mm^3^) was carried out using a Mini-Protean 3 system (Amersham Biosciences, Piscataway, NJ, USA) according to the following procedures: 45 min at a constant power of 5 W, followed by 20 W/gel until the bromophenol blue front reached the bottom of the gel. Subsequently, the proteins in the gels were visualized using the Dodeca silver staining kit (Bio-Rad). The silver-stained protein 2D gels were scanned using an Amersham Biosciences Imagescanner and analyzed using ImageMaster 2D Elite v.6.0 (Amersham Biosciences).

### In-gel digestion and matrix-assisted laser desorption/ionisation-time-of-flightmass spectrometry (MALDI-TOF-MS) identification

Protein spots were excised from gel with an operating knife blade and equilibrated in 50 mM NH_4_HCO_3_ to pH 8.0. After dehydrating with ACN and drying in N_2_ at 37°C for 20 min, the gel pieces were rehydrated in 10 μl trypsin solution (12.5 ng/μl in 50 mM NH_4_HCO_3_) at 4°C for 30 min and incubated at 37°C overnight. Peptides were extracted twice using 0.1% TFA in 30% CAN. The peptides were analyzed using Ultraflex II MALDI-TOF/TOF. Mass spectra were internally and externally calibrated with self-digested fragments of trypsin and standard peptides (Bruker, USA), respectively.

### Protein identification and database searching

Protein identification using peptide mass fingerprinting (PMF) was performed by the Mascot search engine (http://www.matrixscience.com/; MatrixScience Ltd., London, UK) against the SwissProt protein database. The errors in peptide mass were in the range of 100 ppm. One missed tryptic cleavage site per peptide was allowed during the search. Proteins matching more than 4 peptides and with a Mascot score of >64 were considered significant (P<0.05). MH^+^ was selected as the modification. Protein identification results were filtered with the GPS software.

### miRNA target prediction

In order to define the potential targets of miR-27a, 4 publicly available computational algorithms, miRanda, miRWalk, RNA22 and TargetScan, were used. Targets commonly predicted by 2 or more algorithms were considered as putative targets of miR-27a.

### Statistical analysis

The results are representative of multiple experiments and expressed as the means ± SEM. Statistical analysis was performed with an analysis of variance (ANOVA) followed by the Turkey’s t-test. P-values <0.05 denoted statistically significant differences.

## Results

### Stable lentiviral transduction of anti-miRNAs targeting miR-27a in U87 glioma cells

**Figure 1:**
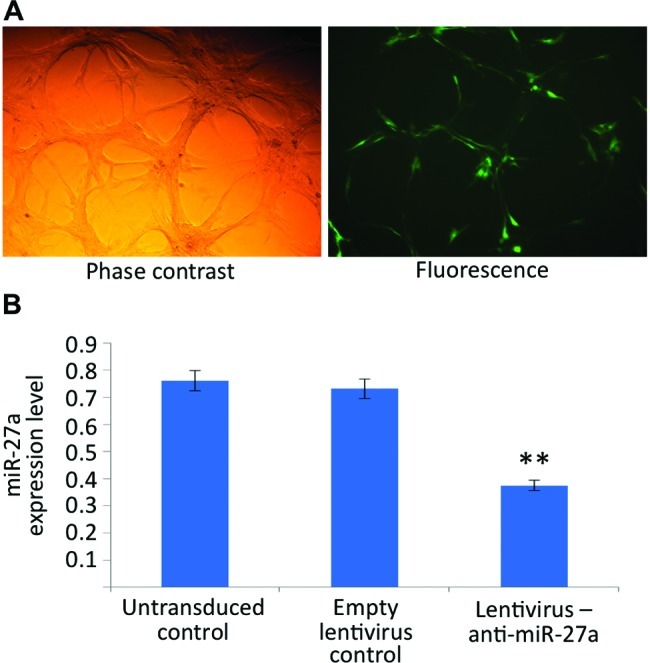
Stable lentiviral transduction of anti-miR-27a into U87 human glioma cells. (A) Transduction efficiency was determined by fluorescent microscopy after transduction with pGreenPuro™ shRNA expression lentivector encoding anti-miR-27a and cop green fluorescence protein (copGFP) for 72 h. Over 80% of transduced U87 cells showed green fluorescent signals. (B) The expression levels of endogenous miR-27a were determined by real-time PCR. The expression of anti-miR-27a was significantly reduced in U87 cells stably transduced with lentivirus encoding anti-miR-27a. ^**^P<0.01, significantly different from both untransduced and empty lentivirus control groups.

The lentivirus transduction efficiency of U87 glioma cells was determined by the detection of GFP signals by fluorescence microscopy at 72 h after transduction and confirmed to be >80% (Fig. 1A). To select stably transduced cells, fluorescence-activated cell sorting was performed. After cell sorting, the miR-27a expression in stably transduced U87 cells was measured by real-time PCR. Fig. 1B demonstrates that the levels of miR-27a were significantly repressed in cells transduced with lentivirus stably expressing anti-miR-27a when compared to the control lentivirus-transduced cells or the untransduced cells.

### Anti-miR-27a reduces the viability and increases apoptosis of U87 cells

**Figure 2:**
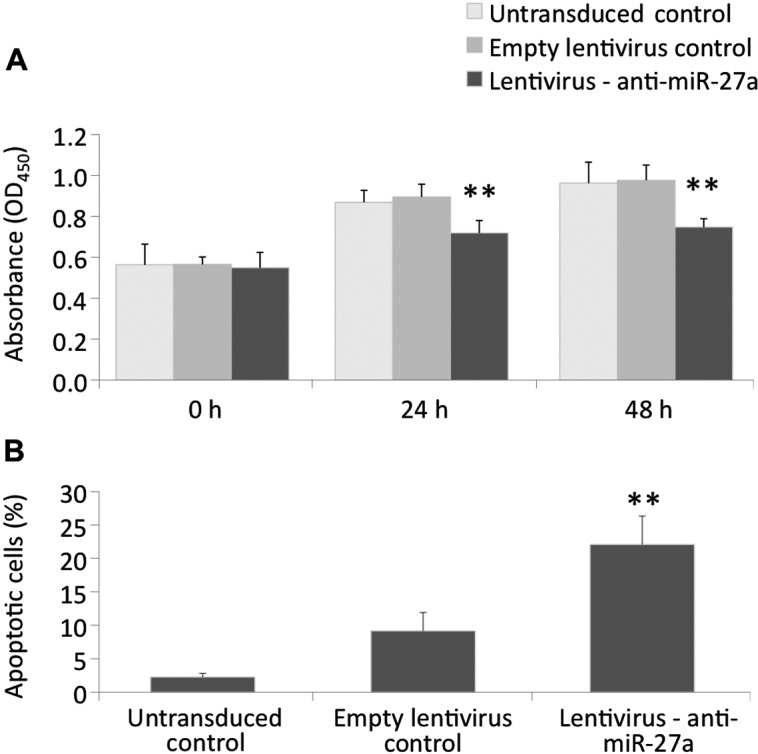
Effects of anti-miR-27a on proliferation and apoptosis of U87 glioma cells. (A) The effect of stable transduction of anti-miR-27a on U87 cell proliferation was determined by the colorimetric Cell Counting kit-8 (CCK-8) assay. Anti-miR-27a significantly impaired the cell proliferation of U87. (B) Apoptotic cells with DNA fragmentation were quantitated as a proportion of the cells in the sub-G1 phase by flow cytometry. Expression of anti-miR-27a substantially induced apoptotic cell death. ^**^P<0.01, significantly different from both untransduced and empty lentivirus control groups.

To study the effect of the blockade of miR-27a on the proliferation of glioma cells, we examined changes in viable cell number by CCK-8 assays after transduction with anti-miR-27a-encoding lentiviral particles in U87 cells. The stable expression of anti-miR-27a significantly impaired the proliferation of U87 cells as indicated by the reduced viable cell number at the 24- and 48-h time-points when compared to the untransduced control or cells transduced with the control lentiviral particles (Fig. 2A). At the 48-h time-point, the viable cell number was significantly reduced by 16.5% when compared to the control lentivirus-transduced group. To further confirm the anti-proliferative action of anti-miR-27a, cell cycle analysis by flow cytometry was performed. The results showed that the stable expression of anti-miR-27a induced a substantial accumulation of U87 cells at the sub-G1 phase without affecting the distribution of other phases of the cell cycle, suggesting that miR-27a inhibition may cause apoptotic cell death in U87 cells (Fig. 2B).

### Anti-miR-27a reduces invasiveness of U87 cells

**Figure 3:**
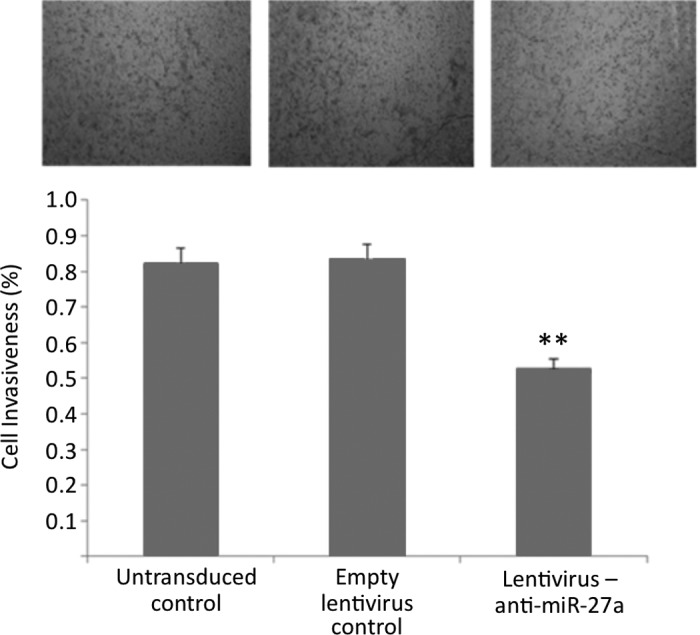
Effect of anti-miR-27a on the invasiveness of U87 glioma cells was determined by Matrigel Transwell assays. Micrographs were shown to indicate the number of cells invaded through the Matrigel membrane from upper chambers to lower chambers in different treatment groups. ^**^P<0.01, significantly different from both untransduced and empty lentivirus control groups.

To investigate the effect of anti-miR-27a expression on cell invasiveness, Transwell invasion assay was performed using a chamber coated with a thin layer of extracellular matrix. The results showed that the stable expression of anti-miR-27a substantially reduced the invasiveness of U87 glioma cells, as indicated by a marked decrease in the number of invaded cells (Fig. 3).

### Target identification by 2D-gel electrophoresis/mass spectrometry

**Figure 4:**
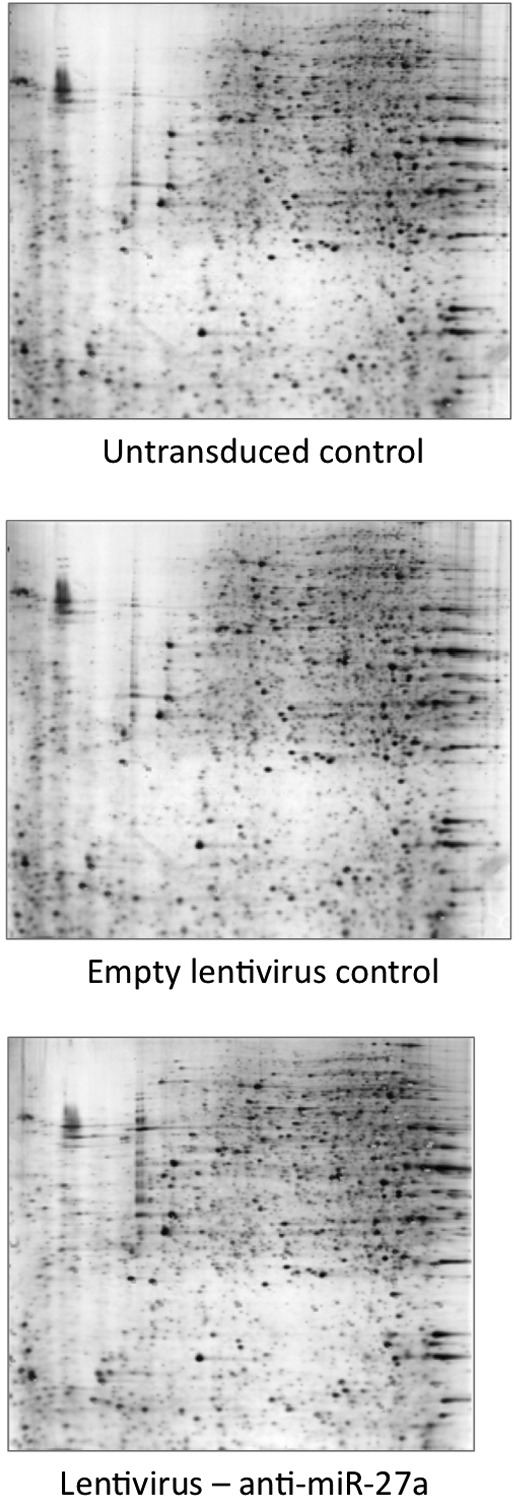
Differential protein expression was determined by comparative gel-based proteomics. Proteins were resolved by isoelectric focusing (IEF) (pH 3–10) in the first dimension (horizontal) followed by SDS-PAGE in the second dimension (vertical). Differentially expressed proteins are shown as black points.

To determine the change in protein profile in response to miR-27a inhibition, gel-based comparative proteomic analysis was performed. As shown in Fig. 4, 29 protein spots were found to be significantly altered in the cells stably expressing anti-miR-27a as compared to the control lentivirus-transduced group, among which 13 and 16 proteins were found to be significantly downregulated and upregulated, respectively. Most of the protein spots of interest were successfully identified by MALDI-TOF MS and by subsequent comparative sequence search in the Mascot database (Table I). Among the 16 upregulated proteins, the mRNAs of 8 proteins were predicted by at least 2 out of 4 computational algorithms to be the direct targets of miR-27a.

## Discussion

miRNA dysregulation plays an active role in cancer development. In this regard, miR-27a, an oncogenic miRNA overexpressed in many types of cancer, has been reported to promote cell proliferation (19), oncogene-induced transformation (23), metastasis (24) and multidrug resistance (25). miR-27a has also been implicated in the regulation of apoptosis (26), angiogenesis (18) and hormone sensitivity (27). The antagonism of miR-27a function thus represents a novel approach for the treatment of cancer. In the present study, we demonstrate that the inhibition of miR-27a by the stable expression of anti-miRNA significantly suppresses the proliferation and invasiveness of U87 glioma cells. Although miR-27a has been shown to be abundantly expressed in glioma tissues (22), this is the first study to demonstrate the influence of miR-27a on the malignant phenotypes of glioma cells.

miRNA performs its biological functions by repressing the protein translation and/or inducing the degradation of its mRNA targets. To date, a number of genes, including Fas associated protein with death domain (FADD) (26), zinc finger and BTB domain containing 10 (ZBTB10; a Sp1 repressor) (28), Ring1 and YY1 binding protein (RYBP; an apoptotic facilitator) (28), Myt-1 (a cdc2 inhibitor) (18), Forkhead box protein O1 (FOXO1) (29), homeodomain-interacting protein kinase-2 (HIPK2) (30), Sprouty2 (21), prohibitin (19) and F-box/WD repeat-containing protein 7 (Fbxw7) (23), have been identified to be the targets of miR-27a. In the current study, we hypthesized that the repressing effects of miR-27a on its targets would be relieved through the stable expression of anti-miR-27a, leading to the upregulation of its targets. By the gel-based comparative proteomic approach, we identified 16 proteins that were upregulated by more than 10-fold in anti-miR-27a-expressing U87 cells. Using multiple computational algorithms, 8 of these upregulated proteins, including the RAD50 homolog, protein disulfide isomerase family A member 5 (PDIA5), dihydropyrimidinase-like 2 (DPYSL2), A kinase anchor protein 4 (AKAP4), lamin A, PRP19/PSO4 pre-mRNA processing factor 19 homolog (PRPF19), septin 11 and enolase 1, were predicted to be the targets of miR-27a in glioma cells. These findings suggest that the combined use of bioinformatic and proteomic approaches may be an efficient method for the identification of novel miRNA targets.

Some of the newly identified proteins putatively targeted by miR-27a have been implicated in cancer biology. For instance, both the RAD50 homolog and PRPF19 have been shown to mediate DNA repair and to have an impact on cell cycle and apoptosis (31,32). The loss of lamin A has also frequently been observed during tumor progression and may contribute to the disruption of nuclear architecture and chromatin structure, thereby increasing genetic instability (33). Moreover, the expression of DPYSL2 has been reported to be reduced in carcinogen-exposed murine lung tissue (34). Septins, a family of cytoskeleton-related proteins dysregulated in cancer, are also involved in cytokinesis, chromosome segregation, DNA repair, migration and apoptosis, all of which are important to cancer development (35). In addition, glioma tissues have been found to possess lower enolase activity than normal brain tissues (36). The mechanism by which the orchestrated expression of these proteins mediates the anticancer effect of anti-miR-27a in glioma cells, however, warrants further investigation.

Lentiviral vectors are promising for gene therapy applications due to their ability to sustain long-term transgene expression (37). In this study, we demonstrate that the lentiviral vector may be used to deliver specific anti-miRNAs to glioma cells and impair their growth and invasiveness. Since 2002, a number of trials using lentiviral vectors for the treatment of both infectious and genetic diseases have been carried out (37). Our study provides *in vitro* evidence that the lentiviral vector may be used to stably express anti-miRNAs in glioma cells. With the advance of tissue-specific expression control and further understanding of miRNA dysregulation in gliomas, we anticipate that the anti-miRNA-encoding lentivirus will become the latest addition to the armamentarium to fight against gliomas in humans in the near future.

## Figures and Tables

**Table I:** Proteins showing a 10-fold up- or downregulation by lentiviral transduction of anti-miR-27a in U87 cells.

Spot no.	Serial no.	Sequence coverage	Score	Down-/up-regulation	Calculated PI value/nominal mass	Protein description	Function	Algorithms that predicted the protein as target of miR-27a
276	gi|14277700	67%	104	Down	6.81/14905	40S ribosomal protein S12	Ribosomal proteins	
363	gi|149242397	50%	105	Down	8.62/15837	Chain A, crystal structure of RhoGDI E155h, E157h mutant	Negative regulator of Cdc42 activation	
381	gi|62702115	59%	139	Down	6.12/25320	Unknown	Unknown	
392	gi|4506181	59%	118	Down	6.92/25996	Proteasome subunit α type-2	Processing of class I MHC peptides	
400	gi|5453549	55%	109	Down	5.86/30749	Peroxiredoxin-4	Activator of the transcription factor NF-κB	
408	gi|6912328	38%	89	Down	5.53/31444	N(G),N(G)-dimethylarginine dimethylaminohydrolase 1 isoform 1	Nitric oxide generation	
422	gi|119603918	49%	93	Down	6.46/31898	Capping protein (actin filament) muscle Z-line, α 2, isoform CRA_a	Regulation of growth of the actin filament by capping the barbed end of growing actin filaments	
428	gi|4506667	57%	141	Down	5.71/34423	60S acidic ribosomal protein P0	Component of the 60S subunit ribosomal protein	
431	gi|4758638	30%	72	Down	6.00/25133	Peroxiredoxin-6	Regulation of phospholipid turnover, protection against oxidative injury	
446	gi|229359377	61%	159	Down	8.75/27690	ADP-ribosyltransferase 4 (Dombrock blood group)	Catalysis of ADP-ribosylation	
457	gi|92911770	62%	189	Down	6.42/23896	XTP3TPA-transactivated protein 1	Transactivation	
768	gi|113411427	37%	78	Up	12.05/29451	PREDICTED: hypothetical protein LOC642441	Unknown	
846	gi|32364694	71%	173	Up	5.44/41317	p40	Lysosomal membrane proteins	
850	gi|225939	32%	76	Up	6.34/36674	Aldehyde reductase	Reversible conversion of an aldose to an alditol	
909	gi|77736367	51%	99	Up	6.60/37177	Poly(rC)-binding protein 2		
1108	gi|4502101	33%	69	Up	6.57/38918	Annexin A1	Potential anti-inflammatory activity	
1135	gi|12056473	38%	85	Up	6.29/40738	Sialic acid synthase	Generating phosphorylated forms of Neu5Ac and KDN	
1279	gi|15277503	61%	202	Down	5.55/40536	ACTB protein	Cytoskeletal protein; associated with asthenospermia	
1291	gi|13938339	31%	113	Up	9.42/44476	ATP5A1 protein	Regulation of apoptosis and possible involvement in colorectal cancers	
1308	gi|203282367	55%	225	Up	6.99/47350	Chain A, crystal structure of human enolase 1	Catalysis of the conversion of 2-phosphoglycerate (2-PG) to phosphoenolpyruvate (PEP)	miRanda, miRWalk
1335	gi|119626209	48%	194	Up	6.50/49260	Septin 11, isoform CRA_b	Regulator of tumor progression in human malignancies	miRanda, miRWalk, TargetScan
1357	gi|1706611	46%	175	Up	7.26/49852	Elongation factor Tu, mitochondrial	Promotion of GTP-dependent binding of aminoacyl-tRNA to the A-site of ribosomes during protein biosynthesis	
1393	gi|7657381	26%	79	Up	6.14/55603	Pre-mRNA-processing factor 19	Cell survival and DNA repair	miRanda, miRWalk, TargetScan
1416	gi|27436946	42%	204	Up	6.57/74380	Prelamin-A/C isoform 1 precursor	Nuclear stability, chromatin structure and gene expression	miRanda, RNA22
1557	gi|169404695	20%	77	Down	8.00/57091	Chain A, pyruvate kinase M2 is a phosphotyrosine binding protein	Phosphotyrosine-binding protein	
1727	gi|21493039	30%	177	Up	6.68/94811	A-kinase anchor protein 4 isoform 2	Regulation of sperm motility	miRanda, miRWalk, TargetScan
1888	gi|308818195	36%	192	Up	5.94/74027	Dihydropyrimidinase-related protein 2 isoform 1	Neuronal differentiation and axonal guidance	miRanda, TargetScan
1994	gi|2687853	22%	80	Up	6.19/155341	RAD50 homologue hsRAD50	Component of the MRN protein complex	miRanda, miRWalk, TargetScan
2055	gi|1710248	24%	88	Up	4.95/46512	Protein disulfide isomerase-related	With isomerase and protein 5 chaperone activities	miRanda, miRWalk, TargetScan
